# Phenomic Diversity and Population–Province Variation in Aromatic Coconut in Southern Thailand Revealed by On-Farm Survey

**DOI:** 10.3390/biology15171513

**Published:** 2026-09-03

**Authors:** Chandrasekhar Manikala, Thanet Khomphet, Noer Rahmi Ardiarini, Chandra Kurnia Setiawan, Pijug Summpunn

**Affiliations:** 1 School of Agricultural Technology and Food Industry, Walailak University, Nakhon Si Thammarat 80160, Thailand; chandrasekharmanikala003@gmail.com; 2 Herbology Research Center, Walailak University, Nakhon Si Thammarat 80160, Thailand; 3 Faculty of Agriculture, Brawijaya University, Malang 65145, Indonesia; rahmi.fp@ub.ac.id; 4 Department of Agrotechnology, Faculty of Agriculture, Muhammadiyah University of Yogyakarta, Kasihan, Bantul 55183, Indonesia; chandra_fp@umy.ac.id; 5 Food Technology and Innovation Research Center of Excellence, School of Agricultural Technology and Food Industry, Walailak University, Nakhon Si Thammarat 80160, Thailand; pijug.su@wu.ac.th

**Keywords:** *Cocos nucifera*, on-farm conservation, BLUPs, phenotypic diversity, Nam Hom coconut, multivariate analysis

## Abstract

Coconut is an important livelihood and industrial crop for coastal communities in Thailand; however, limited information is available on the phenotypic diversity of traditional aromatic coconut populations cultivated by smallholders in southern Thailand. An on-farm survey was conducted in Phang Nga, Trang, Krabi, and Nakhon Si Thammarat, evaluating 27 palms representing nine populations (three palms per population) for 28 quantitative morphological, reproductive, fruit, yield, and coconut-water quality traits. A hierarchical linear mixed model, with province treated as a fixed effect and populations nested within province, was used to characterize phenotypic variation and obtain adjusted population-level BLUPs. Substantial phenotypic variation was observed among the surveyed populations. Var7 in Krabi recorded the highest fruit weight (2039 g) and kernel thickness; Var6 in Trang had the highest number of fruits per bunch (13.3); Var2 in Phang Nga had the highest water volume (430 mL); and Var9 in Nakhon Si Thammarat had the highest number of female flowers (20). Principal component analysis showed that the first five components explained 72.6% of the total phenotypic variation, with fruit, reproductive, water, and vegetative traits contributing strongly to population differentiation. Correlation network analysis further identified coordinated associations among vegetative vigor, leaf morphology, and fruit and yield traits. The study provides a baseline phenotypic characterization of Nam Hom coconut populations under smallholder conditions and identifies population–province combinations with promising trait profiles for further evaluation.

## Simple Summary

Coconut is an important crop for farmers and coastal communities in Thailand, providing food, income, and raw materials for many products. However, limited information is available on how traditional aromatic coconut populations differ when they are grown under different smallholder farming conditions in southern Thailand. This study evaluated nine Nam Hom coconut populations from four provinces for differences in plant growth, leaf and flower characteristics, fruit size, yield-related traits, and coconut water quality. The results showed considerable differences among the populations. Some populations produced heavier fruits, more fruits per bunch, or larger volumes of coconut water, while others showed greater plant height and stronger stem and leaf development. Statistical analyses showed that fruit, water, reproductive, and plant growth characteristics were important sources of the observed diversity. Several population–province combinations showed promising characteristics, including high fruit weight, high coconut water volume, and greater reproductive performance. These populations can therefore be prioritized for further research and conservation. However, the results reflect the specific farms and conditions where the palms were surveyed and should not yet be used to recommend particular varieties to farmers. Larger studies across more locations and seasons are needed to confirm whether these promising characteristics remain consistent and to determine their value for future coconut improvement.

## 1. Introduction

In a world of escalating climatic and agronomic challenges, the complex web of phenotypic diversity in coconut (*Cocos nucifera* L.) germplasm represents an important resource for understanding variation, resilience, and breeding potential. Coconut is cultivated in more than 93 countries worldwide and plays an important role in supporting livelihoods, food and nutritional security, and industrial value chains, particularly in coastal and island regions [1,2]. In 2020, Thailand had a total area under coconut plantations of 137,510.2 ha, with the central region having the largest share of 70,048.32 ha followed by the southern region (58,712.32 ha). Coconut production in these regions is predominantly based on smallholder farming systems, where local populations are cultivated under diverse edaphic and climatic conditions [3].

Phenotypic diversity assessment is fundamental to the effective utilization of germplasm, allowing the identification of promising genotypes, selection of potential parental lines, and the development of specific programs for crop improvement as proposed in the world strategy [4,5]. Morphological characterization is viewed as an inexpensive yet valid proxy measure of genetic variation, especially in perennial crops where the use of molecular technology can be limited by resource supply. Earlier research has shown great diversity in vegetative, reproductive, and fruit component characteristics across conserved coconut accessions [6,7]. For instance, a study conducted at the International Coconut Genebank for South Asia and the Middle East evaluated 16 tall and dwarf coconut genotypes and reported significant genotypic variation in stem girth, bunch number, and nut yield [7]. Through principal component analysis (PCA) and cluster analysis, they determined three broad categories, and the first two components accounted for more than 52 percent of the total variance and indicated strong relationships between fruit weight and copra content. On the same note, Perera et al. [8] evaluated 96 ex-situ accessions of Sri Lanka Tall and reported a high heritability of nut yield (H_b_^2^ = 0.78) and fruit components, where PCA also revealed the differentiation between yield-efficient and high copra quality types. These studies highlight the importance of multivariate methods in the breakdown of multi-factor trait interactions and in providing information on selection indices.

Genetic variability within coconut populations arises from its monoecious nature and predominantly cross-pollinating reproductive biology. Tall cultivars, which constitute most global plantings, exhibit prolonged male and female phase overlap, fostering heterozygosity and accumulating allelic diversity over generations [9,10]. This heterogeneity manifests in a broad phenotypic spectrum, from compact dwarfs suited for high-density planting to vigorous talls valued for timber and sustained yield. However, environmental influences can mask underlying genetic signals, necessitating robust analytical frameworks that partition variance and reveal stable trait associations. By reducing dimensionality and visualizing trait covariation, these methods enable breeders to prioritize key descriptors, detect pleiotropic effects, and construct selection indices that maximize genetic gain [11,12].

In spite of these advances, a regional gap persists in the phenotypic characterization of coconut germplasm, especially in Thailand, where commercial hybrids prevail within plantation sectors, but traditional types in the southern provinces like Phang Nga, Trang, Nakhon Si Thammarat and Krabi are still untapped reservoirs of adaptive phenotypes under varying agroecological conditions and aspects: the coastal sandy loams, the lateritic uplands, and the flood-prone lowlands, each imposing distinct selection pressures on local populations. This knowledge gap has limited the formulation of province-specific breeding objectives, the identification of climate-resilient genotypes, and the design of effective in situ/on-farm conservation strategies. The current study addresses this gap by surveying nine varieties across four provinces and evaluating 28 quantitative traits encompassing vegetative vigor, reproductive characteristics, fruit morphology, and yield-related attributes. A mixed-effects analytical framework was subsequently used to account for variation among survey locations and to estimate variety-level differences in the observed traits [7,8,13,14]. The study evaluated how varietal phenotypes vary across distinct local production environments and identified trait patterns that are consistently observed or potentially associated with particular locations. The main objectives were to: (1) characterize phenotypic variation and divergence among Nam Hom coconut varieties across four southern Thai provinces based on vegetative, reproductive, fruit, and yield-related traits; (2) identify conserved and location-associated trait syndromes using multivariate analyses; (3) estimate variance components and commonly used genetic parameters, including broad-sense heritability and genetic advance as a percentage of the mean (GAM); and (4) rank sampled varieties based on BLUP-derived adjusted performance and develop selection indices to identify promising varieties or populations for further evaluation under specific production environments. The resulting information provides a field-based characterization of phenotypic diversity and varietal performance that can support future replicated multi-environment trials, conservation strategies, and subsequent breeding-oriented evaluation.

## 2. Materials and Methods

### 2.1. Experimental Location and Details

The survey-based experiments were carried out on multiple fields of farmers in four provinces in southern Thailand, namely Trang (7°38′13.6″ N 99°28′31.5″ E), Phang Nga (8°34′24.2″ N 98°15′15.9″ E), Krabi (8°20′02.4″ N 99°03′33.2″ E), and Nakhon Si Thammarat (8°38′49.6″ N 99°51′54.8″ E). The study sites were selected to capture variation in local agro-environmental conditions across the Thai Peninsular region. Although the study region is broadly characterized by a tropical climate, local differences in rainfall distribution, soil properties, topography, elevation, and coastal influence create distinct production environments across locations. The average rainfall is 1800–2800 mm annually, with the main wet season (May–Nov) and the dry period (Jan–Mar) being very short. According to Thai metrological station data from (Dec 2024–Mar 2025) given in the Supplementary Table S1, indicate that the dry-month precipitation is 4.8–106.0 mm (except extreme 1036.4 mm in Nakhon Si Thammarat, during December), mean temperature 26.4–27.2 °C (Dec–Feb–Mar) and increasing humidity 73.9 to 90.5 percent, and light winds (2.1 to 5.5 knots). These four regions exhibit different agro-climatic conditions and vary in soil types and characteristics. The soils consist primarily of Ultisols and Oxisols (sandy clay loam to clay); Trang and Nakhon Si Thammarat have well-drained acidic clay loams (pH = 4.8, EC = 1.2–25 dS m^−1^), whereas Phang Nga has coastal alluvial soils (pH 5.57–6.2), and Krabi has shallow and gravelly clay over limestone soils (pH = 5.0–5.8) [15].

### 2.2. Experimental Details and Plant Materials

The study screened a total of nine Nam Hom coconut varieties, including a combination of commercially cultivated varieties and the local selections that are widely planted in southern parts of Thailand. The mature bearing plants (8–12 years old) of the varieties were randomly selected in existing orchards maintained under the usual management of the farmer, and this made the total number of plants 27 (9 varieties, 3 replications and 4 provinces). The distribution of varieties across the provinces was given in Table 1. Although age uniformity could not be ensured due to the survey nature, only palms within this defined productive age range were included to minimize age-related bias. Var1–Var9 were identified based on farmer-recognized varietal names and available planting-source or field-history information and their phenotypic characteristics. Molecular authentication was not performed; therefore, these groups are referred to as “sampled” or “farmer-recognized” varieties rather than “genetically-authenticated” genotypes.

This study was conducted as an observational on-farm survey rather than a controlled experiment. There were no artificial treatments and interventions applied, and the study captured natural variation under farmer-managed conditions. Therefore, mixed-effects models were used to account for the hierarchical structure of the sampled palms and variation among survey locations. Because the same varieties were not uniformly represented across all provinces, the analysis was not intended to estimate a formal genotype × environment interaction.

### 2.3. Morphological and Yield Trait Assessment

Each plant was assessed using a total of 28 traits that included morphological, reproductive, yield, and quality traits. Vegetative characters comprising plant height (base to apex with measuring tape, cm), stem diameter (30 cm above ground, mm), stem diameter at upper zone, internode length (average of 5 internodes, cm), leaf characteristics such as leaf midrib length, petiole length, and width (average of 3 fully grown leaves of plant, in cm), and number of leaflets, leaflet length, and width. Reproductive parameters (*n* = 3) such as inflorescence length (cm), spathe length and width (cm), and female flowers per inflorescence; fruit set percentage (number of fruits that set/number of flowers). The yield characteristics (*n* = 3) were number of fruits per plant, individual fruit weight (kg, with a digital scale), water volume (mL), and kernel thickness (mm, with calipers). All measurements were done using standard protocols based on the International Plant Genetic Resources Institute (IPGRI) descriptors of tropical fruits [16,17]. Data entries were made in three entries (where necessary, i.e., in case of fruit subsamples) to reduce measurement errors. All observations were collected during a single sampling period (December 2024–March 2025), representing one production cycle under farmer-managed conditions.

### 2.4. Coconut Water Analysis

Mature coconuts (11–12 months after pollination) were harvested from each selected palm. A total of 3 nuts from each palm were collected and immediately stored under cool conditions. The coconut water was filtered through the Whatman No. 1 filter paper and then stored in clean glass beakers. The amount of total soluble solids (°Brix) of the filtered coconut water was determined in triplicate using a digital refractometer (ATAGO PAL-1, Tokyo, Japan). A calibrated pH meter (Mettler Toledo FiveEasy, Greifensee, Switzerland) was used to measure the pH in triplicate and electrical conductivity (EC, mS cm^−1^) in triplicate with a calibrated conductivity meter (Mettler Toledo FiveEasy, Greifensee, Switzerland) [18].

### 2.5. Statistical Analysis

Data were analyzed using R software (version 4.3.1). Descriptive analyses and graphical visualization were performed using appropriate R packages, including psych, ggbiplot, circlize, Agricolae and other supporting packages. For each trait, a linear mixed-effects model was fitted using restricted maximum likelihood (REML), with province treated as a fixed effect and variety nested within province and plant nested within variety-within-province treated as random effects. The model was specified as

$$Y_{ijk}=\mu +P_{i}+V{\left(P\right)}_{j\left(i\right)}+e_{ijk}$$

where $Y_{ijk}$ is the observed trait value, μ is the overall mean, $P_{i}$ is the fixed effect of the ith province, $V{\left(P\right)}_{j\left(i\right)}$ is the random effect of the jth variety nested within the $i_{th}$ province and $e_{ijk}$ represents the residual variation among plants within the corresponding variety-within-province group.

Variance components were estimated by REML. Because varieties were not independently represented across all provinces, the variety and province effects were not completely separable; therefore, the variance component associated with variety-within-province was interpreted as variation among the sampled variety-within-province groups rather than as an unbiased estimate of purely genetic variance. Variance-component-based coefficients of variation were calculated from the REML estimates. The phenotypic variance was defined as

$$\sigma_{ph}^{2}=\sigma_{V\left(P\right)}^{2}+\sigma_{e}^{2}$$

where $\sigma_{V\left(P\right)}^{2}$ is the variance component associated with variety nested within province and $\sigma_{e}^{2}$ is the residual variance.

The variety-within-province coefficient of variation (GCV) was calculated as

$$GCV=\frac{\sqrt{\sigma_{V\left(P\right)}^{2}}}{\bar{X}}\times 100$$

where $\bar{X}$ is the overall trait mean.

The phenotypic coefficient of variation (PCV) and the residual coefficient of variation (ECV) were calculated as

$$PCV=\frac{\sqrt{\sigma_{V\left(P\right)}^{2}+\sigma_{e}^{2}}}{\bar{X}}\times 100$$

$$ECV=\frac{\sqrt{\sigma_{e}^{2}}}{\bar{X}}\times 100$$

A variance-component-based broad-sense heritability ratio was calculated as

$$H_{V\left(P\right)}^{2}=\frac{\sigma_{V\left(P\right)}^{2}}{\sigma_{V\left(P\right)}^{2}+\sigma_{e}^{2}}$$

Because variety and province were partially confounded in the sampling design, this parameter was interpreted as the proportion of phenotypic variance attributable to the sampled variety-within-province component rather than as an independent estimate of genetic heritability. Genetic advance (GA) under a selection intensity of 5% was estimated using a selection differential constant of

$$GA=k\sqrt{\sigma_{V\left(P\right)}^{2}+\sigma_{e}^{2}}\cdot H_{V\left(P\right)}^{2}$$

Genetic advance as a percentage of the mean (GAM) was calculated as

$$GAM=\frac{GA}{\bar{X}}\times 100$$

These parameters were interpreted as variance-component-based indicators of the magnitude of variation and potential response to selection within the sampled variety-within-province structure, rather than as definitive estimates of transmissible genetic variance.

Correlation analysis (Pearson’s) was conducted to explore relationships among traits [19]. Principal component analysis was performed to identify key traits contributing to variation among varieties and provinces [20,21]. For each variety-within-province group, adjusted predictions of the random effects were obtained from the REML-fitted mixed model using BLUPs. The corresponding fixed province effects were estimated using best linear unbiased estimates (BLUEs) [14]. Because province and variety were not independently replicated across all environments, the BLUPs were interpreted as adjusted performance estimates of the sampled variety-within-province groups under the observed field conditions. Similarly, the province BLUEs were used to quantify the systematic differences associated with the sampled provincial environments. Accordingly, the BLUPs should be regarded as model-adjusted predictions that account for the fixed province effect and the hierarchical sampling structure, rather than as direct estimates of breeding value.

## 3. Results and Discussion

### 3.1. Phenotypic Characterization

The phenotypic assessment of nine coconut varieties (Var1–Var9) revealed substantial phenotypic variability in vegetative, reproductive, fruit, and yield-related traits, with coefficients of variation (CV) ranging from less than 10% for traits such as spathe length (SL), number of leaflets (NFL), and fruit length (FL) to more than 40% for plant height (PH), petiole thickness (PT), and kernel thickness (KTH).

#### 3.1.1. Vegetative Characteristics

Substantial variation was observed among vegetative characteristics of the surveyed coconut populations (Table 2). Plant height ranged from 176.33 ± 10.97 cm to 795.00 ± 18.03 cm (CV = 39.43%), indicating significant differences in canopy architecture among the surveyed populations. Within Phang Nga, Var3 exhibited significantly greater plant height and stem dimensions than Var1 and Var2. In Trang, Var4 displayed taller palms than Var5 and Var6, whereas the Krabi population (Var7 and Var8) showed comparatively similar plant stature. Stem diameter at the base ranged from 70.90 ± 0.53 cm to 139.50 ± 1.05 cm (CV = 21.96%), while upper stem diameter varied from 58.70 ± 2.26 cm to 87.43 ± 1.05 cm (CV = 13.02%). Significant within-province differences were detected for several stem-related traits, reflecting phenotypic differentiation among populations under local growing conditions. Leaf-related traits also exhibited notable variation. Midrib length ranged from 257.40 ± 6.48 cm to 384.67 ± 3.16 cm, petiole length from 100.37 ± 0.76 cm to 142.23 ± 0.95 cm, and leaflet length from 83.73 ± 3.14 cm to 113.57 ± 1.10 cm. In Phang Nga, Var2 showed the greatest midrib length, whereas Var3 exhibited relatively longer petioles. Within Trang, Var6 was characterized by greater leaflet length compared with the other populations. In contrast, leaf midrib diameter and number of leaflets showed comparatively lower variability across populations. The findings revealed that quantitative variations among genotypes and these differences could be a result of the wide range of collection sites and possible preferential collection or human selection of various traits. Varieties like Var3 exhibit the greatest plant height and stem girth and good leaf architecture. According to the studies of Sudha et al. [7], the number of leaves and leaf length are significant traits that support palm yield and plant architecture due to their ability to support photosynthesis. These findings align with Sudha et al. [7], Perera et al. [8], and Sudha et al. [22].

#### 3.1.2. Reproductive, Yield, and Coconut-Water Physicochemical Traits

The degree of variation was moderate to high on floral and inflorescence attributes (Table 3). The average number of female flowers (NFF) was (range: 7.67 ± 1.53–20.00 ± 3.00, CV = 28.49), with the highest value observed in Var9 (20.00 ± 3.00), indicating greater female flower production under the observed field conditions. The number of fruits per bunch (NFB; range: 5.00 ± 1.00–13.33 ± 1.53, CV = 30.68) and number of unopened inflorescences (UOI; range: 5.67 ± 0.58–4.00 ± 1.00, CV = 10.54) were used to show consistent floral clustering but still possible improvements in the retention of fruits. Var6 bears the highest number of fruits per bunch compared to the other varieties. Inflorescence length (INL) was relatively uniform (INL range: 61.50 ± 1.18–83.33 ± 2.35 cm, CV = 8.96%), whereas spathe length (SL range: 75.07 ± 2.01–81.97 ± 2.04 cm, CV = 3.20%) and spathe width (SW range: 14.77 ± 2.93–12.17 ± 0.40 cm) exhibit less variability.

Fruit and yield characteristics exhibited a mix of both high and low variability; fruit weight (FW; range: 1283.20 ± 55.38–2039.53 ± 8.64 g) was an important indicator of harvestable yield, with the peak in Var7 and the lowest in Var1, respectively, which are directly correlated with commercial potential. Fruit length (FL; range: 22.87 ± 2.05–26.33 ± 1.00 cm, CV = 5.29%) and fruit diameter (FD; range: 52.67 ± 1.00–43.93 ± 2.57 cm, CV = 6.57%) did not exhibit high variability, implying conserved fruit size and morphology. Pericarp thickness (PTH; range: 22.47 ± 4.74–19.57 ± 0.70 cm, CV = 4.43%) was very homogeneous, and it helped in achieving uniform protection of fruits. Conversely, moderate to high variation was found in spathe width (SW; range: 14.77 ± 2.93–12.17 ± 0.40 cm, CV = 6.95%) and water volume (WV; range: 433.33 ± 68.07–215.00 ± 5.00 mL, CV = 22.26%), and there was a difference between fruit hydration and pulp content. Hole spacing (HS; range: 8.13 ± 0.31 mm–10.17 ± 0.31 mm, CV = 8.03%) was quite stable, whereas kernel thickness (KTH; range: 6.00 ± 0.20–1.00 ± 0.10 cm, CV = 49.22%) varied extremely and had implications for seed size, germination, and processing quality. Additional coconut water characteristics included water pH, which ranged from 4.70 ± 0.52 to 6.42 ± 0.05 (CV = 9.38%), and water electrical conductivity (EC), which ranged from 5.12 ± 0.02 to 7.04 ± 0.03 mS cm^−1^ (CV = 9.67%). The pH values indicated generally acidic coconut water, while variation in EC reflected differences in the ionic composition of coconut water among varieties. Among the reproductive and yield-related traits, Var7 exhibited the highest fruit weight, whereas Var8 recorded the highest fruit diameter. Var2 showed the maximum water volume and hole spacing. Var9 produced the highest number of female flowers per inflorescence, while Var6 recorded the highest number of fruits per bunch. Collectively, these populations may represent useful germplasm resources for future breeding and evaluation programs targeting fruit weight, tender-nut water volume, and bunch productivity. However, additional multi-location and multi-seasonal evaluations with larger sample sizes are required before definitive recommendations can be made regarding their breeding value or broader adaptation. The observed variation in vegetative and reproductive characteristics was accompanied by substantial differences in fruit weight, fruit diameter, and water volume among the surveyed populations. Similar associations between plant morphology and yield-related traits have been reported in coconut germplasm studies by Singh et al. [23].

### 3.2. Phenotypic Variation and Estimation of Genetic Parameters

Analysis of variance for the 28 quantitative traits revealed substantial phenotypic variation among the surveyed coconut populations (Table 3). Province effects were highly significant (*p* < 0.001) for most vegetative, reproductive, and fruit-related traits, indicating a strong influence of local growing conditions on trait expression. Similarly, significant variation among varieties nested within provinces was observed for many traits, including plant height, stem dimensions, leaf characteristics, fruit diameter, fruit weight, water volume, and water electrical conductivity. To further examine these differences, mean comparisons were conducted among varieties within each province. In Phang Nga, significant differences were observed among Var1, Var2, and Var3 for several vegetative and fruit-related traits, particularly plant height, fruit weight, fruit diameter, and water volume. Within Trang, Var4, Var5, and Var6 differed significantly in traits such as fruit weight, leaflet dimensions, and reproductive characteristics. In Krabi, Var7 and Var8 showed contrasting performance for fruit size, kernel thickness, and water quality traits. These within-province comparisons provide statistically valid assessments of phenotypic differentiation under the nested survey design. The trend highlights the fact that there is high genetic variability, especially in vegetative, structural, yield, and quality traits, and thus can be regarded as suitable for breeding programs in terms of targeted selection. Similarly, these findings are correlated with studies of Sudha et al. [7], Perera et al. [8], and Subramanyam et al. [24]. The mean squares were substantially larger for PH, representing high genetic control and low environmental noise. As depicted by the present study, these findings align with recent studies of Odufale [11] and Sudha et al. [25] on coconut germplasm that found that the variation in vegetative, fruit weight, and water-related phenotypes was very highly significant.

Variance components estimated from the mixed-effects models revealed substantial variation among traits (Table 4). Because the present study was conducted as an observational on-farm survey, and the sampled groups were associated with different provinces. Accordingly, the derived coefficients are presented as measures of between-group and phenotypic variation rather than as direct estimates of genetic variation. For most traits, the phenotypic coefficient of variation (PCV) was slightly higher than the corresponding sampled-group coefficient, indicating that residual and environmental sources contributed to the observed phenotypic variation. The differences were particularly small for plant height (46.19% vs. 46.39%), stem diameter at base (25.33% vs. 25.39%), leaflet length (10.66% vs. 10.77%), petiole thickness (45.22% vs. 45.65%), and fruit weight (17.75% vs. 17.97%), whereas larger differences occurred for kernel thickness (25.72% vs. 63.88%), sweetness (4.18% vs. 8.08%), water volume (21.44% vs. 23.83%), and water electrical conductivity (8.85% vs. 11.10%). These patterns indicate that the relative contribution of within-group and residual variation differed substantially among traits.

Residual coefficients of variation (ECV) were generally low for several morphological and fruit traits, including leaflet length (1.58%), stem diameter at base (1.70%), midrib length (4.83%), petiole length (4.25%), leaflet width (4.47%), petiole thickness (6.22%), inflorescence length (3.43%), fruit diameter (2.96%), and fruit weight (2.78%), indicating relatively consistent measurements within the sampled groups. Water volume (10.42%) and water electrical conductivity (6.70%) also showed comparatively moderate residual variation. However, ECV represents residual variability in the present survey and should not be interpreted as a direct estimate of environmental variance.

The proportion of total model-estimated variance attributable to the sampled-group component was high for several traits, including plant height (0.992), stem diameter at base (0.996), stem diameter at the upper zone (0.919), leaf scar spacing at base (0.939), leaflet length (0.979), petiole thickness (0.981), midrib length (0.895), petiole length (0.885), inflorescence length (0.827), fruit diameter (0.818), fruit weight (0.976), and water volume (0.809). These values indicate strong differentiation among the sampled groups; however, genetic effects cannot be independently separated from location, management, and other environmental sources of variation in the current design. Conversely, lower sampled-group variance proportions were observed for number of bunches (0.246), spathe length (0.289), spathe width (0.131), kernel thickness (0.162), and sweetness (0.267), indicating comparatively greater within-group or residual variation. Intermediate proportions were observed for leaf midrib diameter (0.562), number of fruits per bunch (0.599), hole spacing (0.642), water pH (0.745), and electrical conductivity (0.636).

The magnitude of between-group variation was particularly notable for plant height, petiole thickness, stem diameter at base, number of female flowers, number of fruits per bunch, kernel thickness, and water volume, identifying these traits as useful candidates for phenotypic characterization and selection of contrasting palms for subsequent controlled evaluation. Nevertheless, these results do not establish additive genetic control or predict genetic gain. Confirmation of heritable differences would require progeny testing, replicated multi-environment trials, or complementary molecular characterization to disentangle genetic effects from environmental variation. These findings are consistent with recent coconut germplasm studies showing substantial phenotypic and genetic differentiation among tall, dwarf, and hybrid materials while emphasizing the importance of replicated and multi-environment evaluation for reliable breeding inference [7,11,23,26].

### 3.3. Phenotypic Correlation and Network Analysis

**Figure 1:**
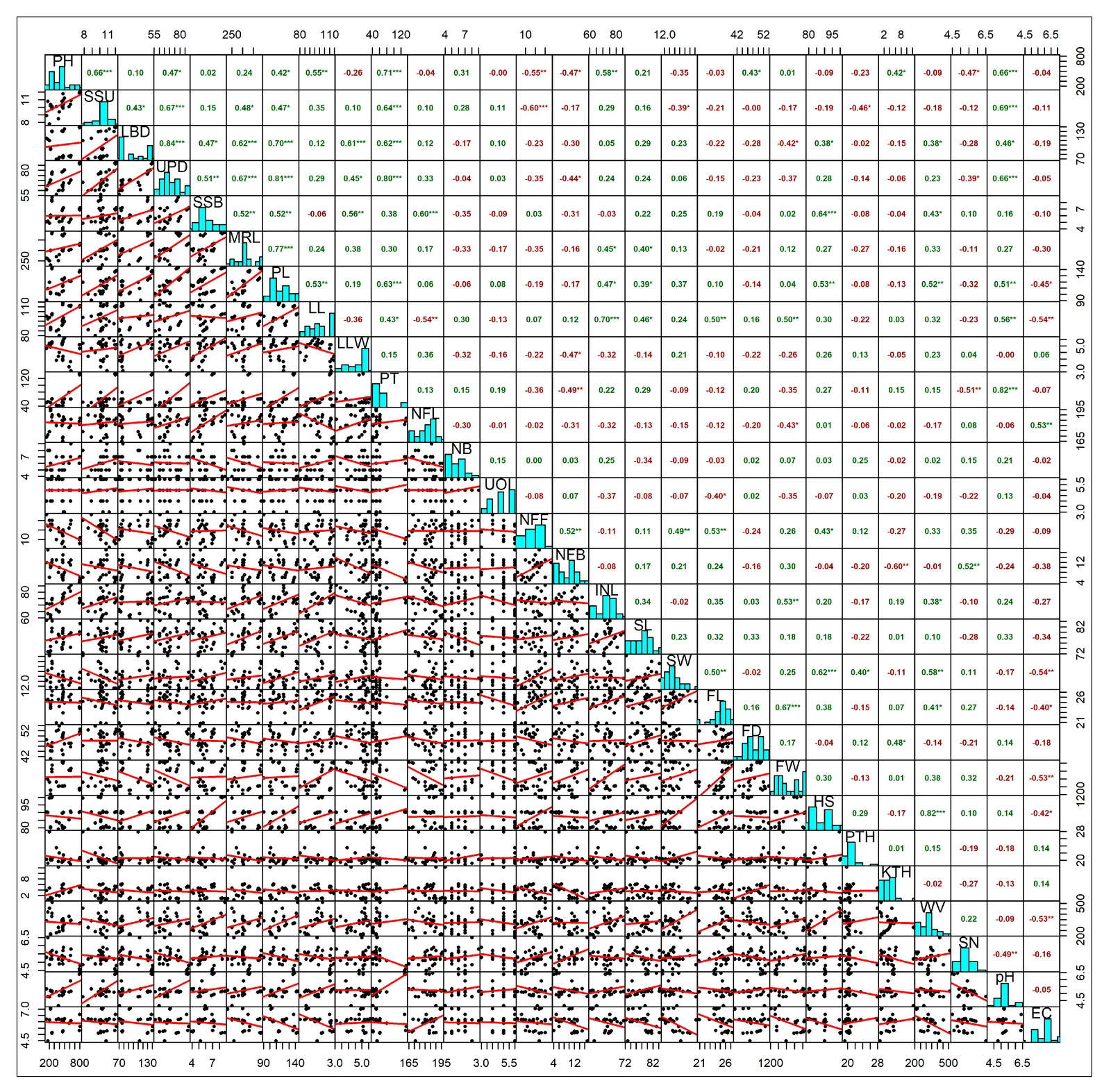
Correlation matrix of 28 quantitative traits in Nam Hom coconut germplasm. Green and red cells indicate positive and negative Pearson correlations, respectively; intensity and size of circles are proportional to correlation coefficients (*** *p* < 0.001, ** *p* < 0.01, * *p* < 0.05). PH: Plant height, LMD: Leaf midrib diameter, LBD: Stem diameter at base, UPD: Stem diameter at upper zone, SSB: Leaf scar spacing at base, MRL: Midrib length, PL: Petiole length, LL: leaflet length, LLW: Leaflet width, PT: Petiole thickness, NFL: Number of leaflets, NFF: Number of female flowers, NB: Number of bunches, UOI: Number of unopened inflorescences, NFB: Number of fruits per bunch, INL: Inflorescence length, SL: Spathe length, SW: Spathe width, FL: Fruit length, FD: Fruit diameter, FW: Fruit weight, HS: Hole spacing, PTH: Pericarp thickness, KTH: Kernel thickness, SN: Sweetness, WV: Water volume, pH: Water pH, EC: Water electrical conductivity.

Phenotypic correlation analysis among the 28 quantitative traits revealed numerous strong and highly significant associations that reflect coordinated expression of vegetative, reproductive, and yield components according to Figure 1. The most significant inter-correlations were found in vegetative traits, and this indicates strong structural integration. There are positive correlations between stem diameter at base (1.5 cm) and stem diameter at upper zone (LBD-UPD; 0.84 ***), along with petiole length and stem diameter at upper zone (PL-UPD; 0.81 ***), and petiole thickness and stem diameter at upper zone (0.80 ***), indicating that taller palms generally possess more robust stems and larger leaf architecture [7]. Some positive correlations exceeding (0.70) were recorded, such as petiole length and stem diameter at base (PL-LBD; 0.70 ***), similarly with petiole length and mid rib length (PL-MRL; 0.77 ***), and petiole thickness with plant height (PT-PH; 0.71 ***). These findings indicate that larger and more robust palms will always have a thicker stem, longer petioles, and a better-built support system of leaves [7,22]. Strong positive correlations include (UPD-LMD; 0.67 ***), (MRL-LBD; 0.62 ***), and (PT-LMD; 0.64 ***), revealing correlations between leaf architecture traits and structural traits. Moderate correlations were recorded, such as plant height with inflorescence length (INL-PH; 0.58 **), leaflet length and plant height (LL-PH; 0.55 **), leaflet width and leaf scar spacing at base (LLW-SSB; 0.56 **), and (LL-PH; 0.53 **), which also support the idea that bigger leaf area is maintained by, and leads to, strong stem architecture. These patterns are in line with past studies on coconuts that found equally high correlations of structural support and correlations between stem girth and petiole dimensions and plant height [27,28].

Reproductive characteristics and yield characteristics were clustered. Water volume was strongly correlated with hole spacing (0.82 ***), indicating larger nuts with substantially more liquid endosperm. Similarly, correlations between pH and petiole thickness (0.82 ***) and inflorescence length and leaflet length (0.70 ***) indicate there is a positive correlation between vegetative and reproductive traits. Fruit weight is positively correlated with fruit length (FW-FL; 0.67 ***). Notable positive correlations were also observed between pH and several structural traits (pH-UPD; 0.66 ***), (pH-LMD; 0.69 ***), (pH-PH; 0.66 ***), (HS-SW; 0.62), and (HS-SSB; 0.64 ***), suggesting that more vigorous palms produce tender nuts with higher endosperm. Reproductive traits displayed moderate but significant associations, including inflorescence length with plant height (INL-PH; 0.58 **), spathe width with number of female flowers (SW-NFF; 0.49 **), fruit weight with inflorescence length (FW-INL; 0.53 **), water volume with spathe width (WV-SW: 0.58 **), and number of fruits per bunch with number of female flowers (NFB-NFF; 0.52 **), suggesting that vegetative vigor is a positive predictor of reproductive output. There was a significant negative relationship between female flower count and midrib diameter of the leaf (NFF-LMD; r = −0.60), which may have been a manifestation of resource trade-offs between structural investment and flower production in field conditions.

**Figure 2:**
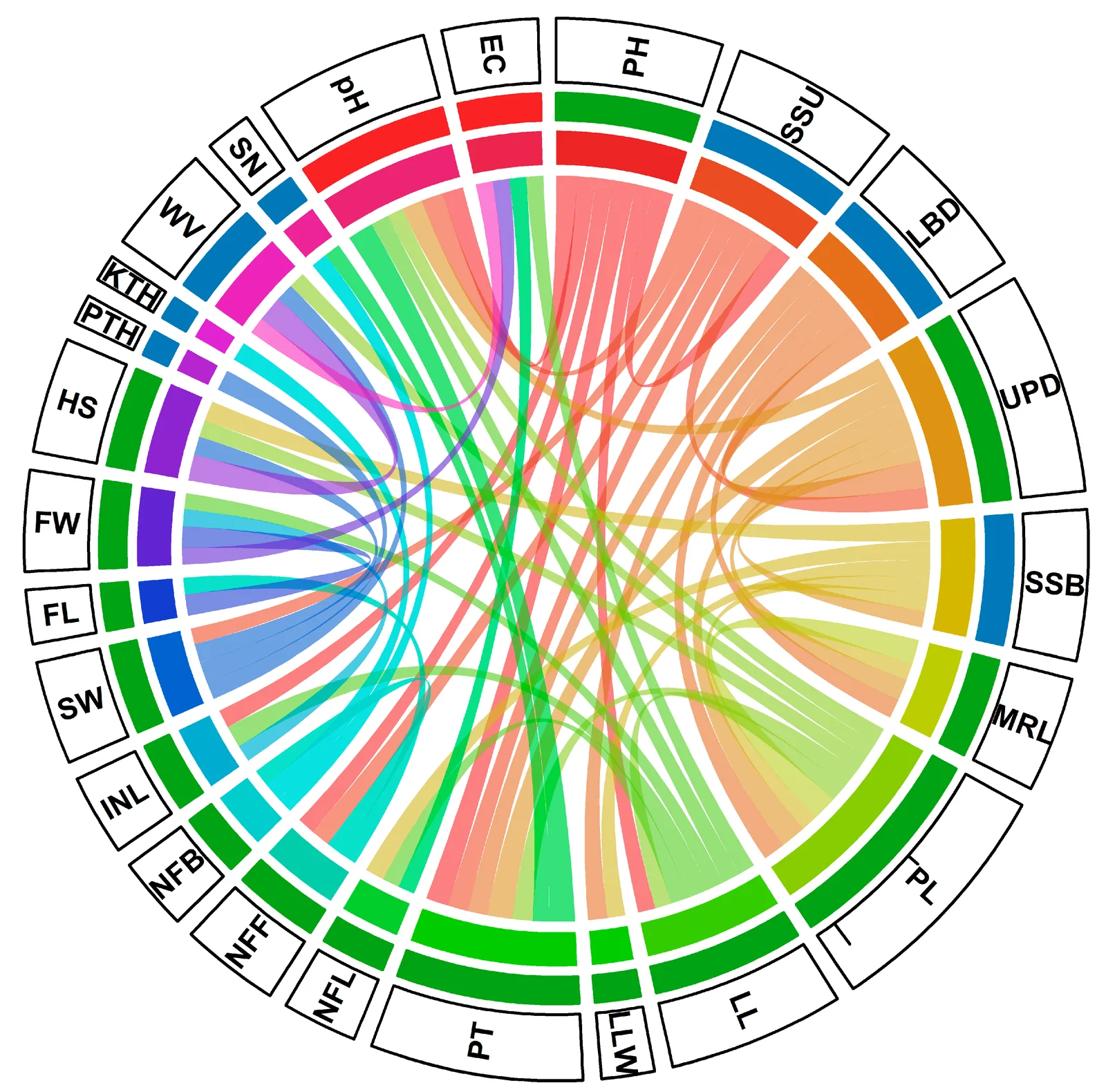
Chord diagram (correlation network) illustrating significant Pearson correlations (|r|≥0.50, p<0.05) among 27 quantitative traits in Nam Hom coconut germplasm. The circular layout groups traits into three major functional syndromes: vegetative vigor, leaf morphology, and nut yield and quality. Trait abbreviations are described in Figure 1.

Correlation network analysis was conducted and represented as a chord diagram (Figure 2), which illustrates three distinct groups of strongly correlated coconut features, each of which is subsequently illustrated by the thickness of the connecting band, indicating strong associations. The nut yield/productivity cluster, which comprises number of bunches (NB), number of female flowers (NFF), number of fruits per bunch (NFB), fruit weight (FW), and water volume (WV), has the largest inter-trait bands, which suggests that there are closely associated reproductive processes, more functional female flowers, and an increase in the number of spikelets or bracts always correlates with increasing nut yield.

Another densely knit network of thick chords, the leaf morphology cluster comprising leaflet length (LL), leaflet width (LLW), number of leaflets (NFL), midrib length (MRL), petiole length (PL), and petiole thickness (PT), indicates that changes in the size of the leaf and the rachis take place. Simultaneously, this is evidence of coordinated vegetative development and canopy structure. Lastly, fruit/nut morphometric clusters such as fruit diameter (FD), fruit weight (FW), hole spacing (HS), and kernel thickness (KTH) display good internal connectivity with fruit size, shell diameter, husk size, and kernel thickness all increasing with each other and drive to the point of integrated nut morphology development. The strong positive correlations of vegetative vigor traits and yield components give a clear genetic basis for indirect selection according to previous studies. Sudha et al. [25] and Anthonio et al. [27] reported that traits like stem diameter, petiole length, or hole spacing can reliably predict fruit weight, water volume, and reproductive potential. These synchronized connections, in line with worldwide coconut germplasm assessments, are a solid indication of how to improve the stature of plants, the leaf structure, or the amount and quality of nuts concurrently during breeding programs focusing on southern Thailand [26].

### 3.4. Principal Component Analysis and Trait Contributions

**Figure 3:**
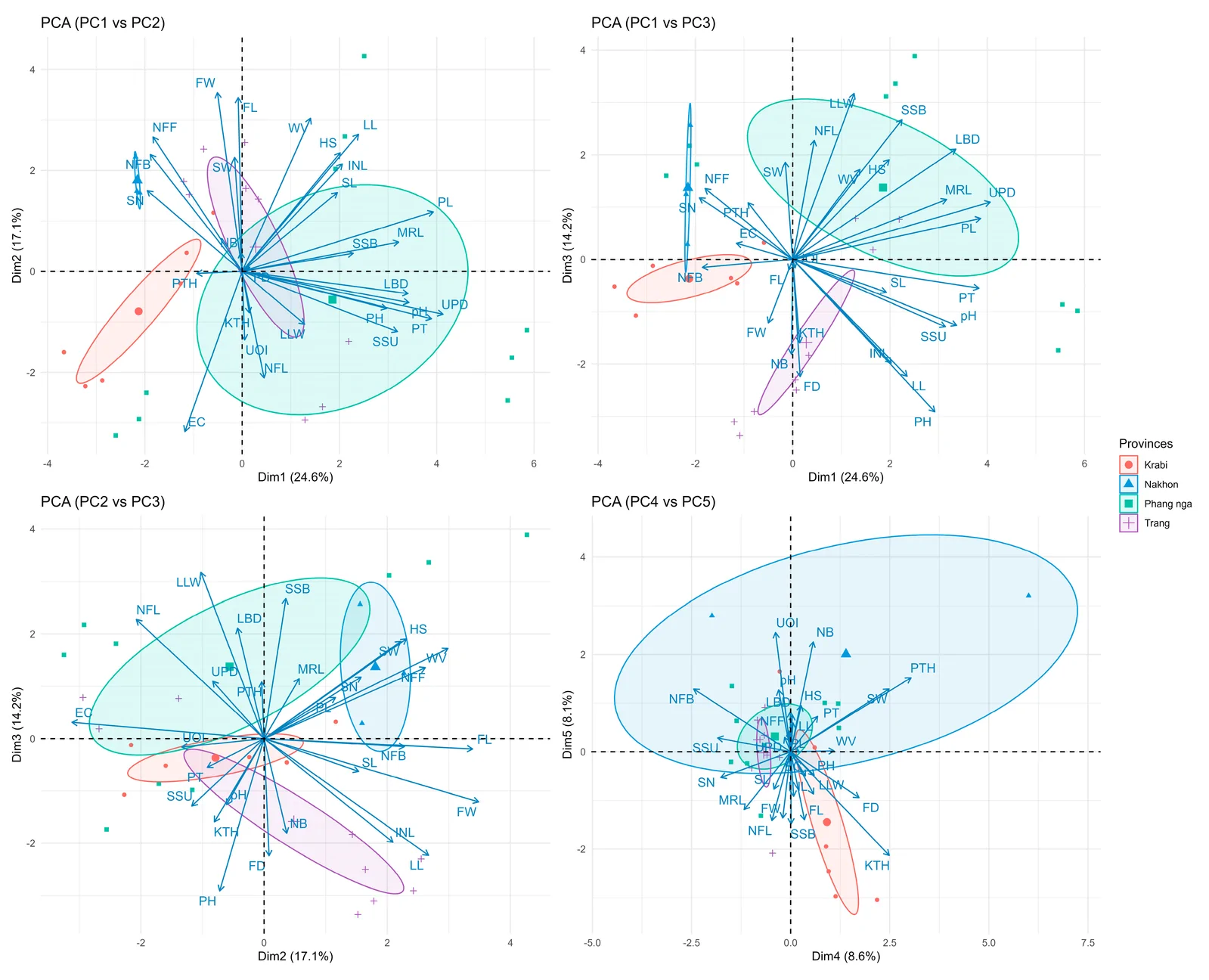
Principal component analysis of nine Nam Hom coconut varieties. The first five principal components explained 72.6% of the total variation (PC1 = 24.6%, PC2 = 17.1%, PC3 = 14.2%, PC4 = 8.6%, and PC5 = 8.1%). Colors and ellipses indicate provincial groupings, while arrows represent trait loadings. Trait abbreviations are described in Figure 1.

The first principal component (PC1) explained 24.6% of the total variation and was primarily associated with yield, fruit, and reproductive characteristics, while the second principal component (PC2) accounted for 17.1% and reflected variation in vegetative and structural traits. Together, PC1 and PC2 explained 41.7% of the total phenotypic variation. The third principal component (PC3) contributed an additional 14.2%, whereas PC4 and PC5 explained 8.6% and 8.1% of the variation, respectively. Collectively, the first five principal components accounted for 72.6% of the total variation, indicating that these components adequately captured the major dimensions of phenotypic diversity present in the coconut populations (Figure 3). This indicates that phenotypic diversity was distributed across multiple dimensions rather than being concentrated in a few dominant traits. Traits showing the strongest positive loadings on PC1 included fruit weight (FW), water volume (WV), fruit diameter (FD), husk thickness (HS), inflorescence length (INL), number of female flowers (NFF), number of fruits per bunch (NFB), and leaflet length (LL), indicating that these traits were major contributors to phenotypic differentiation among populations. PC2, however, was associated with vegetative structural traits (stem diameter at base (SDB), upper stem diameter (UPD), petiole thickness (PT), plant height (PH), and midrib length (MRL)), whereas kernel thickness (KTH) and electrical conductivity (EC) loaded in opposite directions, indicating differential patterns of trait association. At the provincial level, samples from Phang Nga were associated with higher values of fruit weight, water volume, and reproductive traits under the surveyed conditions, while Krabi populations were more closely associated with kernel thickness-related traits. Nakhon Si Thammarat and Trang populations occupied intermediate positions, reflecting diverse trait combinations. These findings are in agreement with the global studies on coconut PCA, wherein the first principal component has been shown to explain between 20 and 30 percent of variance and is explained by fruit weight, water volume, and reproductive features, with the second and third dimensions explaining vegetative vigor and trade-offs between kernels [5,7,26].

### 3.5. Population-Associated Trait Performance and Implications for Future Selection

#### 3.5.1. Provincial Mean Performance and Trait Distribution

**Figure 4:**
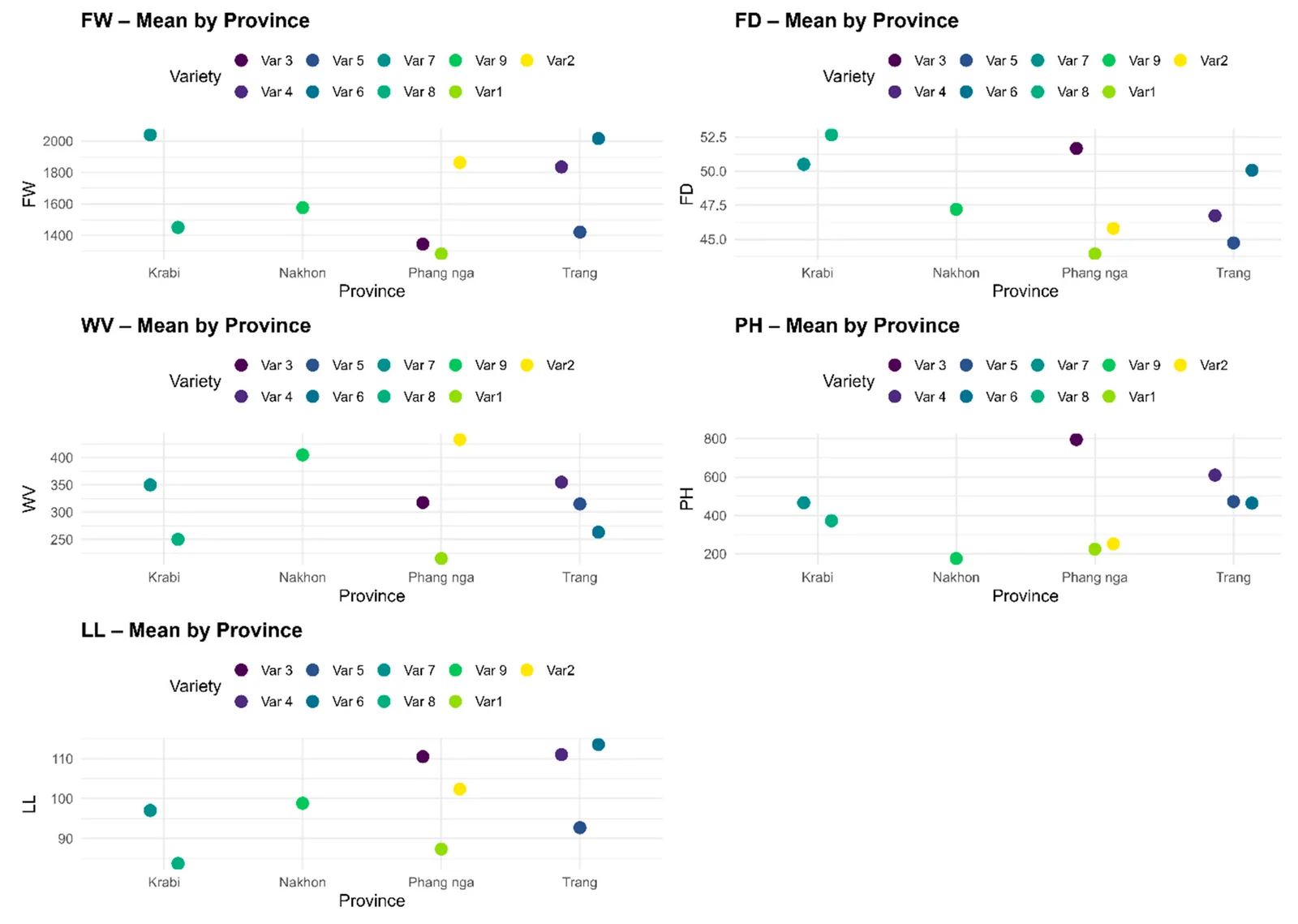
Mean performance of nine Nam Hom coconut varieties for five key commercial traits across four provinces in southern Thailand. Each point represents the mean of three palms per variety (nested design). Trait abbreviations are described in Figure 1.

**Figure 5:**
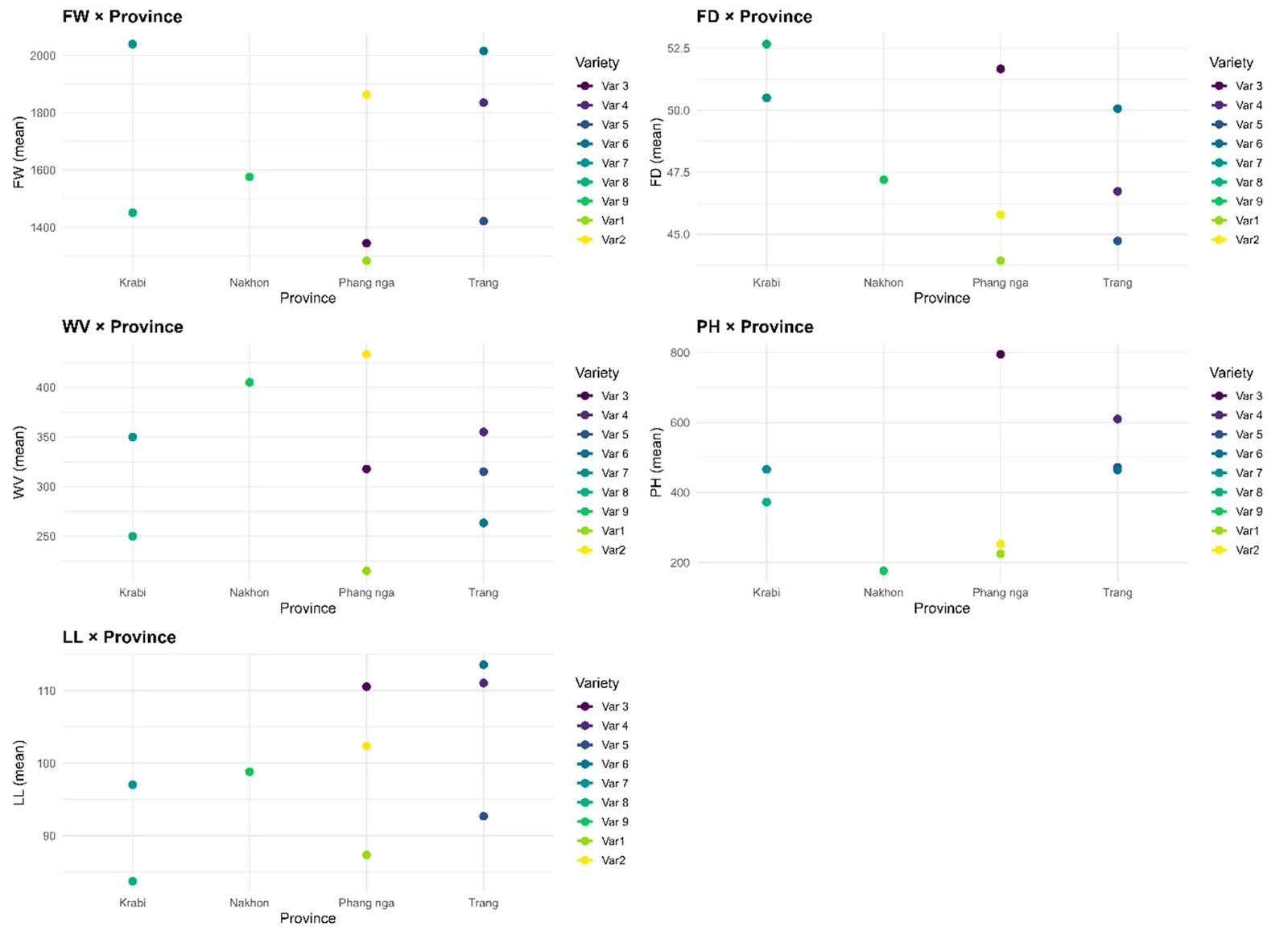
Genotype-by-province interaction plots for five economically important traits in Nam Hom coconut germplasm. Points represent variety means within each province, illustrating strong province-specific expression and adaptation. Trait abbreviations are described in Figure 1.

**Figure 6:**
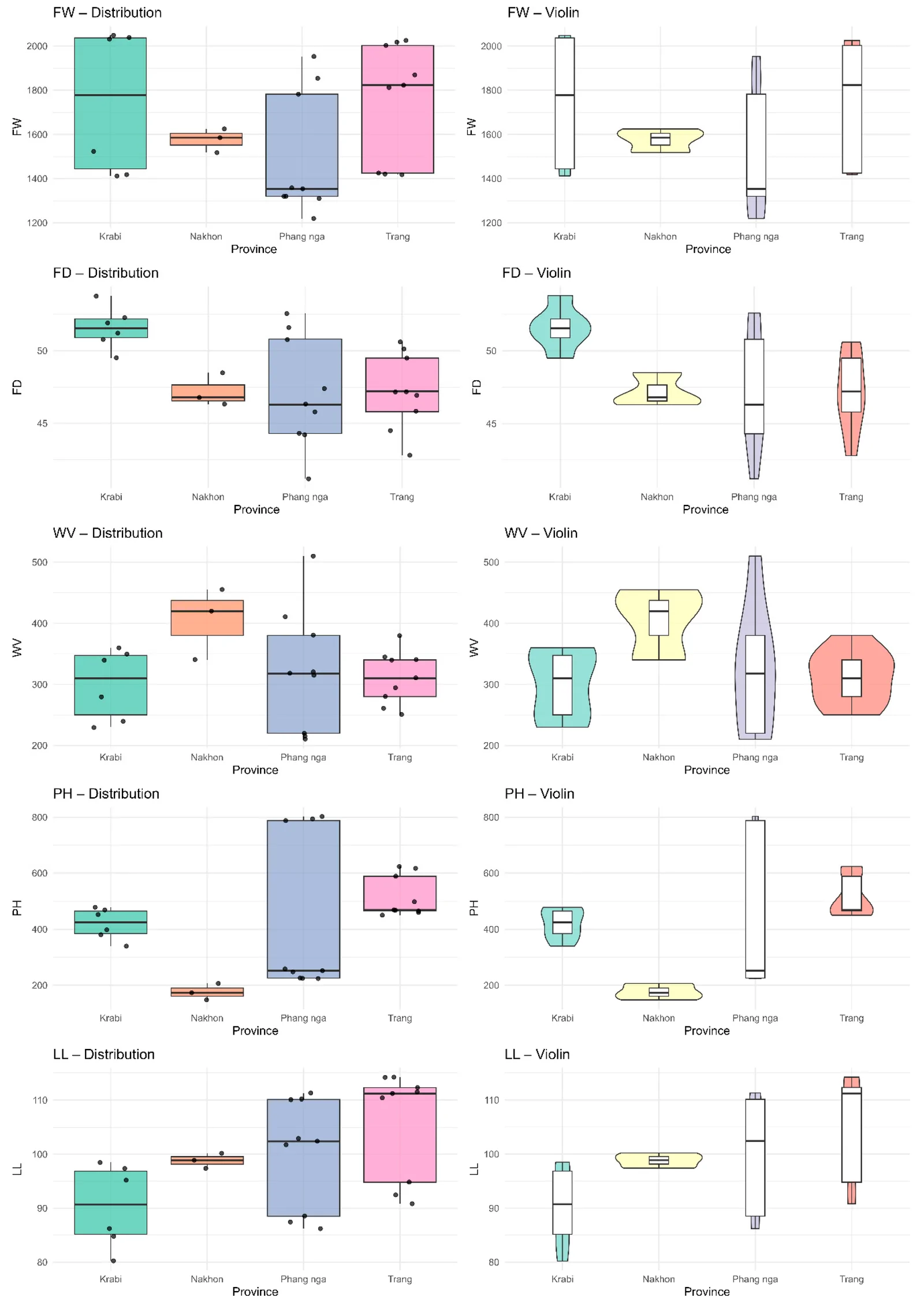
Violin plots showing the distribution of five key commercial traits (fruit weight, fruit diameter, nut water volume, plant height, and leaflet length) across the four provinces in southern Thailand. Phang Nga exhibited the highest and most stable values for fruit and water-related traits. Trait abbreviations are described in Figure 1.

The mean performance of the nine coconut varieties in the four provinces indicated unique territorial benefits in terms of location-specific according to Figure 4 and Figure 5. Phang Nga province distinctly displayed the best yield traits in terms of commercial importance, based on the maximum fruit weight (FW ≈ 1900–2000 g), fruit diameter (FD ≈ 52 cm), nut water volume (WV ≈ 400 mL), and longest leaflet length (LL≈110 cm). In contrast, Krabi and Nakhon provinces favored superior vegetative vigor, with several varieties exceeding 600–700 cm in plant height of the plants (PH). These patterns indicate differences in the observed phenotypic profiles of the populations sampled in the respective provinces, rather than demonstrating provincial effects or superior agro-ecological adaptation. Furthermore, the patterns of trait distribution of the five major economic characteristics in the four provinces indicated significant differences in the mean performance and the phenotypic diversity (Figure 6). Fruit weight (FW), fruit diameter (FD), water volume (WV), and leaflet length (LL) continued to exhibit the largest median and the smallest distributions in Phang Nga (~1900 g, ~52 cm, ~400 mL, and ~105–110 cm, respectively) with a few outliers, which suggests that the environment is highly homogenous and favorable to large, high-water tender nuts ideal for commercial production. Similarly, Trang exhibited the widest spreads, broadest violin shapes, and the most outliers for nearly all traits, which indicates that it has a high degree of phenotypic plasticity, environmental sensitivity, or underlying genetic heterogeneity among sampled palms. Plant height (PH) displayed the opposite pattern, where Krabi and Nakhon Si Thammarat had the tallest palms (medians > 600 cm) and distributions with good consistency, indicating high vegetative vigor in the areas, whereas the Phang nga and Trang palms were shorter and more variable, indicating priority in resource allocation towards fruit rather than stem extension. In general, Phang Nga was the best province in the stability of commercially desirable fruit characteristics, whereas Krabi and Nakhon were good in vegetative growth, probably because of the variations in the depth of the soil, water table, and intensity of the management.

The contrasting patterns between fruit and vegetative traits are, nevertheless, biologically informative. Populations with relatively high fruit weight and water volume may contain phenotypes of interest for tender nut production, whereas populations characterized by greater plant stature may provide useful material for studies of vegetative architecture. However, the observation that taller populations occurred in Krabi and Nakhon Si Thammarat should not be interpreted as evidence of preferential resource allocation toward vegetative growth at the expense of fruit production. Such physiological trade-offs would require measurements of resource availability, biomass allocation, reproductive efficiency, and repeated observations across environments to establish. However, the provincial distributions provide evidence of substantial geographic structuring of observed phenotypic variation among the surveyed aromatic coconut populations. This finding is consistent with previous coconut germplasm studies showing considerable variation in morphological, fruit, and yield-related characteristics among populations and accessions [7,8,22,26,27,29]. These populations should be prioritized for replicated multilocation trials before any recommendation for cultivar deployment or breeding use is made.

#### 3.5.2. BLUP-Based Identification of Promising Coconut Populations for Future Evaluation

The Best Linear Unbiased Predictors (BLUPs) provided adjusted estimates of population-associated performance for the surveyed coconut populations (Table 5 and Table 6). BLUP-based approaches are useful for ranking genotypic effects in mixed-model analyses, particularly in perennial crops where environmental variation can substantially influence phenotypic performance [13,14]. However, because each population was evaluated in only one province, the present analysis cannot distinguish population effects from location effects or estimate genotype × environment interaction. Therefore, the BLUPs are interpreted here as adjusted population-level performance estimates rather than definitive breeding values or measures of agro-ecological adaptation.

Several populations exhibited noteworthy trait combinations. In Krabi, Var7 showed high fruit weight (2039 g), fruit diameter (52.7 cm), kernel thickness (6.0 mm), and water volume (350 mL), whereas Var6 in Trang showed high fruit weight (2016 g) and number of fruits per bunch (13.3). In Phang Nga, Var2 showed high water volume (430 mL), hole spacing (100.2 cm), and fruit weight (1862 g), while Var3 exhibited particularly high plant height (795 cm), stem diameter (139.5 cm), and kernel thickness (5.2 mm). In Nakhon Si Thammarat, Var9 showed a relatively high number of female flowers per inflorescence (20) together with a water volume of 402 mL.

These population-specific trait combinations identify candidate germplasm for further evaluation, particularly for tender-nut, fruit-quality, reproductive, and vegetative traits [30]. However, the observed superiority of individual populations is specific to the surveyed population–province combinations and should not be interpreted as evidence of broad adaptation, stability, or immediate farmer recommendation. Multilocation and multiyear replicated trials are required to validate these phenotypes and to determine whether the observed rankings are maintained across environments. The results therefore provide a preliminary basis for prioritizing populations such as Var7, Var6, Var2, and Var9 for subsequent validation and breeding research rather than designating them as province-specific promising varieties.

### 3.6. Cluster Analysis and Phenotypic Divergence

**Figure 7:**
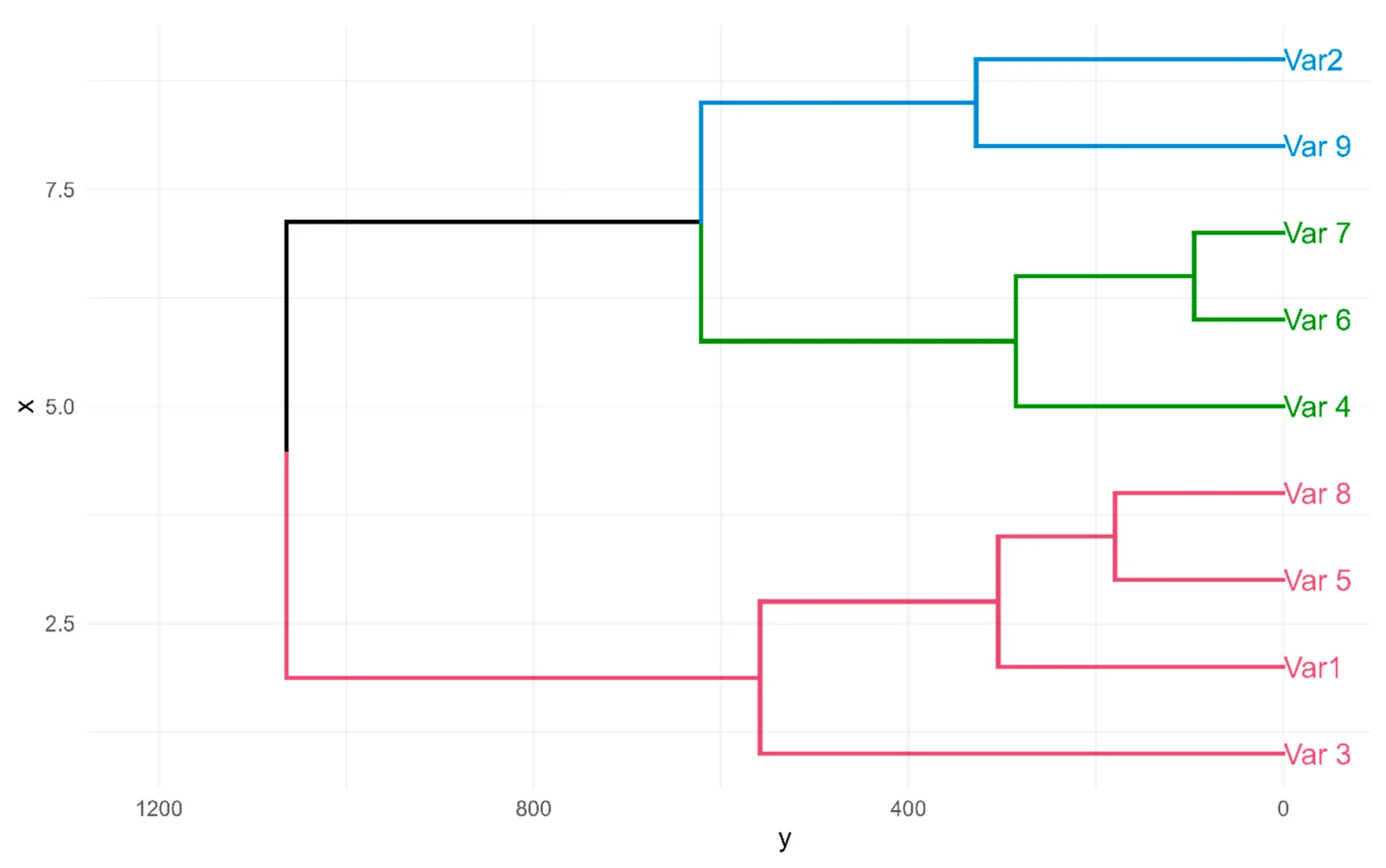
Hierarchical clustering of Nam Hom coconut varieties based on 27 quantitative traits. Three distinct clusters were formed: Cluster 1 green (high yielding: Var4, Var6, Var7), Cluster 2 blue (moderate: Var2, Var9), and Cluster 3 red (vegetatively diverse: Var1, Var3, Var5, Var8).

Hierarchical clustering based on Ward’s method using Euclidean distances computed from the 28 quantitative traits, the 9 coconut varieties were clustered into three separate clusters at about 60% similarity level (Figure 7). Cluster 1 (green) comprises Var4, Var6 and Var7, which are the high-yielding clusters that are characterized by high fruit weight, water volume and reproductive performance. Cluster 2 (blue) includes Var2 and Var9, which had medium expression of vegetative and yield traits. The largest and most diverse Cluster is 3 (red), which includes Var1, Var3, Var5, and Var8 encompassing extremes in plant height (Var3 being the tallest) and the overall morphological diversity. The dendrogram also revealed a close resemblance of Var2 and Var9, and Var3 seemed to be somewhat distant in the cluster, which indicates its exclusivity in the combination of extreme height and structural robustness. The prominent distinction of the high-yielding genotypes (Var4, Var6, and Var7) into a distinct cluster is a clear indication of a significant amount of phenotypic variance that is largely influenced by traits of fruit and reproductive characteristics. These findings are consistent with previous studies [7,24,26]. where Ward’s clustering method effectively grouped coconut genotypes based on yield potential and vegetative vigor. The relatively large inter-cluster distances, particularly between Cluster 1 (high yielding) and Cluster 3 (morphologically diverse), suggest potential for selecting contrasting parental lines in breeding programs aimed at improving yield and fruit quality in southern Thailand.

A key limitation of this study is the relatively small sample size, which may not fully capture intra-varietal variability in a perennial, cross-pollinated crop such as coconut. This is inherent to on-farm survey studies conducted under farmer-managed conditions, where environmental heterogeneity cannot be controlled. In addition, observations were based on a single sampling period, which may not fully reflect seasonal variation in yield and reproductive traits. Future studies should include larger sample sizes, multi-location trials, and repeated seasonal assessments to validate these findings.

## 4. Conclusions

This on-farm assessment revealed substantial phenotypic differentiation among nine Nam Hom coconut populations across four provinces in southern Thailand, particularly for vegetative, reproductive, fruit, and water-related traits. High sampled-group variance ratios were observed for traits such as plant height (0.992), stem diameter at the base (0.996), petiole thickness (0.981), fruit weight (0.976), and water volume (0.809). However, because variety and province were confounded in the survey design, these estimates should be constructed as between-group variation rather than direct evidence of heritable genetic control. Multivariate analyses showed that the first five principal components explained 72.6% of the total phenotypic variation (PC1 = 24.6%, PC2 = 17.1%, PC3 = 14.2%, PC4 = 8.6%, and PC5 = 8.1%), with fruit weight, water volume, fruit dimensions, reproductive traits, and vegetative architecture contributing strongly to population differentiation. BLUP-based comparisons identified promising population–province combinations, including Var7 in Krabi, Var6 in Trang, Var2 in Phang Nga, and Var9 in Nakhon Si Thammarat, for specific yield, fruit, water, or reproductive traits. These populations should be considered candidates for further evaluation rather than confirmed superior or broadly adapted varieties.

## Figures and Tables

**Table 1:** Distribution of Nam Hom coconut varieties across surveyed provinces in Southern Thailand.

Province	Variety Code	Palm Age (Years)	Number of Sampled Palms
Phang Nga	Var1	8	3
	Var2	10	3
	Var3	9	3
Trang	Var4	9	3
	Var5	9	3
	Var6	8	3
Krabi	Var7	9	3
	Var8	8	3
Nakhon Si Thammarat	Var9	8	3

**Table 2:** Morphological, reproductive, yield, and quality traits of 28 traits across nine Nam Hom coconut varieties evaluated in southern Thailand.

Traits	Var1	Var2	Var3	Var4	Var5	Var6	Var7	Var8	Var9
Vegetative Traits									
Plant height (PH)	225.00 ± 1.00 ^c^	252.90 ± 5.03 ^b^	795.00 ± 7.55 ^a^	610.00 ± 18.52 ^b^	472.33 ± 24.17 ^c^	464.33 ± 4.04 ^c^	466.33 ± 13.20 ^c^	372.67 ± 29.69 ^d^	176.33 ± 29.57 ^e^
Leaf midrib diameter (LMD)	10.30 ± 0.10 ^b^	10.00 ± 0.17 ^bc^	12.00 ± 0.10 ^a^	10.47 ± 0.40 ^abc^	11.27 ± 0.50 ^ab^	10.50 ± 0.40 ^abc^	10.13 ± 0.25 ^bc^	9.33 ± 0.80 ^c^	9.17 ± 1.52 ^c^
Stem diameter at base (LBD)	92.27 ± 1.05 ^c^	130.23 ± 0.67 ^b^	139.50 ± 1.05 ^a^	76.03 ± 1.60 ^e^	132.33 ± 2.87 ^b^	76.13 ± 0.70 ^e^	74.47 ± 1.40 ^e^	70.90 ± 2.02 ^e^	112.67 ± 2.52 ^d^
Stem diameter at upper zone UPD)	70.40 ± 0.26 ^c^	77.33 ± 1.01 ^b^	87.43 ± 1.05 ^a^	69.27 ± 1.64 ^cd^	79.40 ± 1.11 ^b^	63.13 ± 2.66 ^de^	64.63 ± 3.48 ^de^	58.70 ± 2.26 ^e^	64.73 ± 3.72 ^de^
Leaf scar spacing at base (SSB)	6.00 ± 0.17 ^c^	8.47 ± 0.31 ^a^	7.03 ± 0.15 ^b^	5.30 ± 0.26 ^cd^	5.57 ± 0.35 ^cd^	4.07 ± 0.15 ^e^	7.03 ± 0.21 ^b^	5.53 ± 0.40 ^cd^	5.07 ± 0.15 ^de^
Midrib length (MRL)	276.93 ± 40.73 ^c^	384.67 ± 3.16 ^a^	329.67 ± 4.07 ^b^	303.47 ± 2.41 ^bc^	375.20 ± 4.88 ^a^	304.53 ± 3.91 ^bc^	302.37 ± 5.28 ^bc^	267.03 ± 15.13 ^cd^	257.40 ± 6.48 ^d^
Petiole length (PL)	102.30 ± 0.95 ^d^	142.23 ± 0.95 ^a^	137.40 ± 1.11 ^ab^	121.30 ± 0.56 ^c^	124.57 ± 1.46 ^bc^	114.07 ± 0.85 ^c^	105.23 ± 0.67 ^d^	100.37 ± 0.76 ^d^	106.47 ± 14.69 ^d^
Leaflet length (LL)	87.37 ± 1.15 ^e^	102.37 ± 0.55 ^bc^	110.53 ± 0.67 ^a^	111.03 ± 0.57 ^a^	92.70 ± 2.01 ^d^	113.57 ± 1.10 ^a^	97.03 ± 1.68 ^cd^	83.73 ± 3.14 ^e^	98.83 ± 1.40 ^c^
Leaflet width (LLW)	5.03 ± 0.12 ^a^	5.27 ± 0.06 ^a^	5.00 ± 0.17 ^a^	3.43 ± 0.35 ^c^	5.40 ± 0.20 ^a^	4.03 ± 0.15 ^b^	5.30 ± 0.20 ^a^	4.20 ± 0.30 ^b^	5.00 ± 0.20 ^a^
Petiole thickness (PT)	50.10 ± 0.26 ^c^	60.47 ± 0.31 ^bc^	132.33 ± 2.34 ^a^	66.70 ± 11.09 ^b^	60.47 ± 0.31 ^bc^	40.97 ± 0.80 ^d^	50.23 ± 0.55 ^c^	41.40 ± 0.62 ^d^	51.20 ± 1.40 ^c^
Number of leaflets (NFL)	195.00 ± 1.00 ^a^	187.33 ± 3.06 ^b^	184.00 ± 3.00 ^bc^	181.33 ± 4.16 ^bcd^	183.67 ± 3.21 ^bc^	167.33 ± 2.08 ^e^	185.00 ± 3.00 ^bc^	186.33 ± 5.51 ^b^	171.33 ± 2.52 ^de^
Reproductive and Yield Characteristics									
Number of female flowers (NFF)	16.67 ± 1.53 ^ab^	18.33 ± 1.53 ^ab^	9.67 ± 1.53 ^c^	18.00 ± 2.00 ^ab^	7.67 ± 1.53 ^c^	13.67 ± 1.53 ^b^	13.00 ± 1.00 ^b^	13.33 ± 3.06 ^b^	20.00 ± 3.00 ^a^
Number of bunches (NB)	5.67 ± 0.58 ^ab^	4.33 ± 0.58 ^b^	6.67 ± 0.58 ^a^	7.33 ± 1.53 ^a^	5.67 ± 1.15 ^ab^	6.33 ± 1.15 ^a^	6.33 ± 0.58 ^a^	5.33 ± 1.53 ^ab^	7.00 ± 1.73 ^a^
Number of unopened inflorescences (UOI)	5.33 ± 1.15 ^a^	5.00 ± 1.00 ^a^	5.67 ± 0.58 ^a^	4.67 ± 0.58 ^a^	4.67 ± 1.53 ^a^	5.00 ± 1.00 ^a^	4.00 ± 1.00 ^a^	5.67 ± 0.58 ^a^	5.00 ± 1.00 ^a^
Number of fruits per bunch (NFB)	10.67 ± 0.58 ^ab^	12.00 ± 1.00 ^a^	5.00 ± 1.00 ^c^	12.00 ± 1.00 ^a^	7.00 ± 1.00 ^bc^	13.33 ± 1.53 ^a^	6.00 ± 1.00 ^bc^	10.33 ± 2.52 ^ab^	11.67 ± 5.51 ^a^
Inflorescence length (INL)	61.50 ± 1.18 ^c^	73.53 ± 2.08 ^ab^	73.50 ± 1.70 ^ab^	83.33 ± 2.35 ^a^	76.87 ± 3.56 ^ab^	75.63 ± 0.67 ^ab^	74.37 ± 2.80 ^ab^	65.47 ± 0.83 ^bc^	68.53 ± 4.46 ^bc^
Spathe length (SL)	75.07 ± 2.01 ^b^	81.97 ± 2.04 ^a^	80.90 ± 2.02 ^a^	77.80 ± 2.88 ^ab^	76.90 ± 2.10 ^ab^	80.50 ± 0.90 ^a^	75.23 ± 1.29 ^b^	78.07 ± 1.47 ^ab^	76.57 ± 6.79 ^ab^
Spathe width (SW)	12.40 ± 0.50 ^b^	14.20 ± 0.36 ^a^	12.67 ± 0.81 ^b^	12.43 ± 0.35 ^b^	12.17 ± 0.40 ^b^	13.17 ± 0.31 ^ab^	12.53 ± 0.40 ^b^	12.53 ± 0.65 ^b^	14.77 ± 2.93 ^a^
Fruit length (FL)	24.23 ± 0.45 ^b^	26.33 ± 1.00 ^a^	24.33 ± 0.68 ^b^	26.03 ± 0.60 ^a^	22.87 ± 2.05 ^b^	26.17 ± 0.31 ^a^	26.43 ± 0.91 ^a^	23.57 ± 2.41 ^b^	25.40 ± 0.20 ^ab^
Fruit diameter (FD)	43.93 ± 2.57 ^c^	45.80 ± 1.60 ^bc^	51.67 ± 0.90 ^ab^	46.73 ± 0.81 ^bc^	44.73 ± 2.06 ^c^	50.07 ± 0.55 ^ab^	50.50 ± 0.89 ^ab^	52.67 ± 1.00 ^a^	47.20 ± 1.15 ^bc^
Fruit weight (FW)	1283.20 ± 55.38 ^cd^	1863.23 ± 85.81 ^ab^	1344.50 ± 20.89 ^cd^	1835.10 ± 30.54 ^b^	1421.47 ± 3.55 ^c^	2015.60 ± 11.36 ^a^	2039.53 ± 8.64 ^a^	1451.27 ± 62.28 ^c^	1576.33 ± 54.10 ^bc^
Hole spacing (HS)	8.37 ± 0.25 ^b^	10.17 ± 0.31 ^a^	9.37 ± 0.25 ^ab^	8.73 ± 3.07 ^b^	8.40 ± 0.95 ^b^	8.17 ± 0.31 ^b^	8.87 ± 0.31 ^b^	8.13 ± 2.67 ^b^	9.33 ± 5.34 ^ab^
Pericarp thickness (PTH)	20.27 ± 0.15 ^a^	20.37 ± 0.25 ^a^	20.43 ± 0.35 ^a^	19.57 ± 0.70 ^a^	20.07 ± 0.51 ^a^	20.07 ± 0.35 ^a^	20.60 ± 0.85 ^a^	21.77 ± 1.92 ^a^	22.47 ± 4.74 ^a^
Kernel thickness (KTH)	1.07 ± 0.15 ^d^	1.00 ± 0.10 ^d^	5.20 ± 0.20 ^a^	3.43 ± 0.35 ^bc^	4.30 ± 0.26 ^ab^	3.37 ± 0.55 ^bc^	6.00 ± 0.20 ^a^	5.43 ± 5.95 ^a^	3.00 ± 2.21 ^c^
Sweetness (SN)	5.43 ± 0.21 ^a^	5.43 ± 0.25 ^a^	4.63 ± 0.15 ^b^	5.43 ± 0.35 ^a^	5.20 ± 0.10 ^a^	5.10 ± 0.17 ^a^	5.67 ± 0.25 ^a^	5.23 ± 0.35 ^a^	5.55 ± 0.85 ^a^
Water volume (WV)	215.00 ± 5.00 ^d^	433.33 ± 68.07 ^a^	317.67 ± 2.52 ^bc^	355.00 ± 21.79 ^ab^	315.00 ± 22.91 ^c^	263.33 ± 15.28 ^cd^	350.00 ± 10.00 ^ab^	250.00 ± 26.46 ^cd^	405.00 ± 58.95 ^a^
Water Ph (pH)	5.20 ± 0.02 ^c^	5.15 ± 0.03 ^c^	6.42 ± 0.05 ^a^	5.38 ± 0.45 ^bc^	5.11 ± 0.03 ^c^	5.31 ± 0.04 ^bc^	4.90 ± 0.05 ^d^	4.70 ± 0.52 ^d^	4.96 ± 0.32 ^cd^
Water electrical conductivity (EC)	7.04 ± 0.03 ^a^	5.12 ± 0.02 ^d^	5.84 ± 0.66 ^bc^	5.99 ± 0.10 ^b^	6.20 ± 0.03 ^b^	5.25 ± 0.04 ^d^	6.06 ± 0.06 ^bc^	6.19 ± 0.03 ^b^	5.61 ± 0.98 ^c^

Note: Values are expressed as Mean ± standard deviation. Means sharing different lowercase letters within the same horizontal row indicate statistically significant differences at *p* < 0.05 according to Duncan’s Multiple Range Test.

**Table 3:** Analysis of variance for 28 traits based on a hierarchical model with provinces and varieties nested within provinces.

Traits	Trait Codes	Province (MS)	Province: Variety (MS)	MSE (Residual)	CV (%)
Plant height	PH	86,487.12 ***	134,589.2 ***	328.55	4.25
Stem diameter at base	SSU	3.15 **	1.83 **	0.4	6.14
Leaf midrib diameter	LBD	3011.98 ***	2021.11 ***	2.93	1.70
Stem diameter at upper zone	UPD	376.01 ***	179.59 ***	4.93	3.15
Leaf scar spacing at base	SSB	8.25 ***	3.28 ***	0.07	4.27
Midrib length	MRL	6227.45 ***	5885.11 ***	225.5	4.82
Petiole length	PL	859.69 ***	611.76 ***	24.77	4.25
leaflet length	LL	285.22 ***	374.33 ***	2.47	1.58
Leaflet width	LLW	1.07 ***	1.61 ***	0.04	4.47
Petiole thickness	PT	1824.15 ***	2646.59 ***	14.67	6.22
Number of leaflets	NFL	339.51 ***	132.36 ***	10.78	1.80
Number of bunches	NB	2.19 ^ns^	2.79 ^ns^	1.3	18.74
Number of unopened inflorescences	UOI	0.54 ^ns^	1.01 ^ns^	0.96	19.63
Number of female flowers	NFF	40.04 ***	57.72 ***	3.89	13.62
Number of fruits per bunch	NFB	12.69 ^ns^	35.59 ***	4.93	22.70
Inflorescence length	INL	167.91 ***	102.05 ***	6.18	3.43
Spathe length	SL	11.23 ^ns^	23.18 ^ns^	8.42	3.71
Spathe width	SW	4.09 *	1.45 ^ns^	1.18	8.35
Fruit length	FL	0.15 ^ns^	8.34 **	1.45	4.80
Fruit diameter	FD	30.41 ***	29.66 ***	2.03	2.96
Fruit weight	FW	128,430.2 ***	337,006.9 ***	2091.23	2.78
Hole spacing	HS	1.87 ***	1.4 **	0.21	5.45
Pericarp thickness	PTH	5.81 ^ns^	0.52 ^ns^	3.11	8.55
Kernel thickness	KTH	13.49 ^ns^	7.37 ^ns^	4.54	58.47
Water volume	WV	8208.25 **	19,852.18 ***	1130.33	10.42
Sweetness	SN	0.17 ^ns^	0.35 ^ns^	0.13	6.91
Water pH	pH	0.84 ***	0.66 ***	0.06	4.85
Water electrical conductivity	EC	0.23 ^ns^	1.43 ***	0.16	6.70

Note: *** Significant at *p* < 0.001, ** Significant at *p* < 0.01, * Significant at *p* < 0.05, ns = Not significant.

**Table 4:** Genetic parameter estimates: genotypic coefficient of variation (GCV), phenotypic coefficient of variation (PCV), broad-sense heritability (H_b_^2^) variance ratio based on the sampled variety-within-province component, and genetic advance as percent of mean (GAM) of traits in Nam Hom coconut.

Trait Codes	Traits	Sampled-Group Variance	Residual Variance	GCV (%)	PCV (%)	ECV (%)	H_b_^2^	GA	GAM (%)
PH	Plant height	38,740.785	328.549	46.193	46.388	4.254	0.992	403.755	94.756
SSU	Leaf midrib diameter	0.520	0.404	6.963	9.284	6.141	0.562	1.114	10.757
LBD	Stem diameter at base	647.981	2.928	25.328	25.385	1.703	0.996	52.320	52.058
UPD	Stem diameter at upper zone	56.009	4.926	10.607	11.063	3.145	0.919	14.781	20.948
SSB	Leaf scar spacing at base	1.022	0.066	16.831	17.365	4.274	0.939	2.019	33.604
MRL	Midrib length	1929.330	225.496	14.112	14.914	4.825	0.895	85.618	27.508
PL	Petiole length	189.970	24.770	11.770	12.514	4.250	0.885	26.705	22.805
LL	Leaflet length	112.820	2.474	10.655	10.771	1.578	0.979	21.645	21.713
LLW	Leaflet width	0.451	0.045	14.167	14.854	4.465	0.910	1.319	27.833
PT	Petiole thickness	774.496	14.666	45.222	45.648	6.223	0.981	56.794	92.287
NFL	Number of leaflets	42.000	10.778	3.554	3.984	1.800	0.796	11.909	6.53
NB	Number of bunches	0.423	1.296	10.706	21.586	18.744	0.246	0.664	10.937
NFF	Number of female flowers	15.735	3.889	27.391	30.590	13.618	0.802	7.317	50.527
NFB	Number of fruits per bunch	7.358	4.926	27.742	35.845	22.699	0.599	4.325	44.23
INL	Inflorescence length	29.624	6.180	7.505	8.250	3.428	0.827	10.199	14.062
SL	Spathe length	3.426	8.415	2.370	4.405	3.714	0.289	2.051	2.626
SW	Spathe width	0.177	1.176	3.239	8.957	8.351	0.131	0.313	2.413
FL	Fruit length	1.273	1.446	4.506	6.586	4.803	0.468	1.590	6.352
FD	Fruit diameter	9.151	2.033	6.283	6.947	2.962	0.818	5.637	11.708
FW	Fruit weight	85,566.473	2091.228	17.752	17.968	2.775	0.976	595.355	36.130
HS	Hole spacing	0.380	0.212	7.303	9.112	5.450	0.642	1.018	12.056
KTH	Kernel thickness	0.878	4.541	25.716	63.880	58.475	0.162	0.777	21.326
WV	Water volume	4785.083	1130.337	21.436	23.834	10.418	0.809	128.163	39.715
SN	Sweetness	0.049	0.134	4.175	8.076	6.914	0.267	0.236	4.445
pH	Water pH	0.188	0.064	8.276	9.591	4.847	0.745	0.771	14.712
EC	Water electrical conductivity	0.275	0.158	8.854	11.104	6.701	0.636	0.861	14.542

**Table 5:** Best linear unbiased predictors of the nine coconut varieties for key commercial traits across four provinces in Southern Thailand.

Province	Variety	Fruit Weight (g)	Nut Water Volume (mL)	Plant Height (cm)	Stem Diameter at Base (cm)	Kernel Thickness (cm)	Fruit Diameter (cm)
Krabi	Var7	**2039** **.** **5**	**349** **.** **9**	466.2	74.5	**6** **.** **00**	**52** **.** **7**
	Var8	1450.4	249.1	371.9	70.9	3.93	52.7
Trang	Var6	**2015** **.** **6**	263.0	464.3	76.1	3.34	50.1
	Var4	1834.9	354.6	**609** **.** **8**	76.0	3.42	46.7
	Var5	1421.5	314.5	471.9	**132** **.** **3**	**4** **.** **30**	44.7
Phang Nga	Var2	**1861** **.** **9**	**429** **.** **9**	252.9	**130** **.** **2**	1.00	45.8
	Var3	1344.4	317.7	**795** **.** **0**	**139** **.** **5**	**5** **.** **20**	**51** **.** **7**
	Var1	1282.4	215.0	225.0	92.3	1.06	43.9
Nakhon Si Thammarat	Var9	1575.7	**402** **.** **0**	174.7	112.6	2.45	47.2

Note: Bold values = highest BLUP in each province for that trait.

**Table 6:** Province-specific ranking of varieties for the most important yield and quality traits (based on BLUPs).

Province	Highest Fruit Weight	Highest Nut Water	Tallest Palm	Thickest Stem Base	Thickest Kernel
Krabi	**Var7** (2039 g)	**Var7** (350 mL)	Var7 (466 cm)	Var7 (74.5 cm)	**Var7** (6.0 mm)
Trang	**Var6** (2016 g)	Var4 (355 mL)	**Var4** (610 cm)	**Var5** (132 cm)	Var5 (4.3 mm)
Phang Nga	**Var2** (1862 g)	**Var2** (430 mL)	**Var3** (795 cm)	**Var3** (139 cm)	**Var3** (5.2 mm)
Nakhon Si Thammarat	Var9 (1576 g)	**Var9** (402 mL)	Var9 (175 cm)	Var9 (113 cm)	Var9 (2.5 mm)

Note: bold values represent the varietal rankings.

## Data Availability

The data that support the findings of this study are available from the corresponding author upon reasonable request.

## Supplementary Materials

The following supporting information can be downloaded at: https://www.mdpi.com/article/10.3390/biology15171513/s1, Table S1: Monthly climatic data (December 2024–March 2025) obtained from the Thai metrological department in the four surveyed provinces of southern Thailand.
