# Supplementary material for: Phenomic Diversity and Population–Province Variation in Aromatic Coconut in Southern Thailand Revealed by On-Farm Survey
Source: Biology (Basel). 2026 Sep 3;15(17):1513. doi: 10.3390/biology15171513 (PMC13565545; doi:10.3390/biology15171513)
Supplement: Supplementary file 1 [file biology-15-01513-s001.zip › biology-4518462-supplementary.pdf]

Supplementary Table S1. Monthly climatic data (December 2024-March 2025) obtained from the Thai metrological department in the four surveyed provinces of southern Thailand.

ID	Station	Avg WS. (knot)	Avg Temp.(°C)	Acc Prec. (mm)	Avg Pres. (hPa)	Avg Humi. (%)
December (2024)	Trang Weather Observing Station	3.8	27.0	37.4	1,006.4	-
December (2024)	Krabi Weather Observing Station	5.2	26.5	36.2	1,006.0	86.1
December (2024)	Phang Nga Weather Observing Station	2.3	27.2	45.8	1,009.1	79.4
December (2024)	Nakhon Si Thammarat Weather Observing Station	2.1	26.5	1,036.4	1,008.5	90.5
January (2025)	Trang Weather Observing Station	4.4	26.9	36.2	1,007.2	-
January (2025)	Krabi Weather Observing Station	5.5	26.7	4.8	1,006.8	78.5
January (2025)	Phang Nga Weather Observing Station	2.6	27.2	9.0	1,009.8	73.9
January (2025)	Nakhon Si Thammarat Weather Observing Station	2.4	26.4	200.4	1,010.4	85.3
February (2025)	Trang Weather Observing Station	4.1	27.9	106.0	1,007.6	-
February (2025)	Krabi Weather Observing Station	3.6	27.7	54.6	1,007.2	75.6
February (2025)	Phang Nga Weather Observing Station	2.6	27.5	37.4	1,010.3	77.1
February (2025)	Nakhon Si Thammarat Weather Observing Station	2.7	27.2	103.4	1,008.9	81.8
March (2025)	Trang Weather Observing Station	3.6	28.6	51.6	1,006.5	-
March (2025)	Krabi Weather Observing Station	3.1	28.5	65.4	1,004.1	77.5
March (2025)	Phang Nga Weather Observing Station	2.2	28.3	74.8	1,009.2	80.0
March (2025)	Nakhon Si Thammarat Weather Observing Station	2.5	28.3	72.6	1,009.5	82.1
